# An essential developmental function for murine phosphoglycolate phosphatase in safeguarding cell proliferation

**DOI:** 10.1038/srep35160

**Published:** 2016-10-12

**Authors:** Gabriela Segerer, Kerstin Hadamek, Matthias Zundler, Agnes Fekete, Annegrit Seifried, Martin J. Mueller, Frank Koentgen, Manfred Gessler, Elisabeth Jeanclos, Antje Gohla

**Affiliations:** 1 Institute of Pharmacology and Toxicology, University of Würzburg, Versbacher Strasse 9, D-97078 Würzburg, Germany; 2 Rudolf Virchow Center for Experimental Biomedicine, University of Würzburg, Josef-Schneider-Strasse 2, D-97080 Würzburg, Germany; 3 Institute of Pharmaceutical Biology, University of Würzburg, Julius-von-Sachs-Platz 2, D-97082 Würzburg, Germany; 4 Ozgene Pty Ltd, PO Box 1128, Bentley DC, WA 6983, Australia; 5 Theodor-Boveri-Institute/Biocenter, Developmental Biochemistry, Am Hubland, University of Würzburg, D-97074 Würzburg, Germany; 6 Comprehensive Cancer Center Mainfranken, University of Würzburg, Josef-Schneider-Strasse 6, D-97080 Würzburg, Germany

## Abstract

Mammalian phosphoglycolate phosphatase (PGP) is thought to target phosphoglycolate, a 2-deoxyribose fragment derived from the repair of oxidative DNA lesions. However, the physiological role of this activity and the biological function of the DNA damage product phosphoglycolate is unknown. We now show that knockin replacement of murine *Pgp* with its phosphatase-inactive *Pgp*^*D34N*^ mutant is embryonically lethal due to intrauterine growth arrest and developmental delay in midgestation. PGP inactivation attenuated triosephosphate isomerase activity, increased triglyceride levels at the expense of the cellular phosphatidylcholine content, and inhibited cell proliferation. These effects were prevented under hypoxic conditions or by blocking phosphoglycolate release from damaged DNA. Thus, PGP is essential to sustain cell proliferation in the presence of oxygen. Collectively, our findings reveal a previously unknown mechanism coupling a DNA damage repair product to the control of intermediary metabolism and cell proliferation.

Aerobic life favours the formation of endogenous and exogenous reactive oxygen species that constantly cause DNA damage^1^,^2^,^3^,^4^. This naturally occurring, oxidative DNA damage affects both the nucleobases and 2-deoxyribose and causes a broad spectrum of DNA lesions, which can contribute to carcinogenesis, neurodegeneration and aging^1^. While mechanisms of DNA damage repair are being unravelled, much less is known about the fate and the biological effects of DNA damage products that are released from DNA^5^.

The free radical-initiated fragmentation of the DNA 2-deoxyribose sugar moiety generates single and double strand DNA breaks with a 3′-phosphate or a 3′-phosphoglycolate (3′-PG) terminus^2^,^3^. Because these 3′-ends preclude direct DNA re-ligation, they need to be processed to enable DNA repair. Enzymes such as tyrosyl DNA phosphodiesterase-1 (TDP1) cleave 3′-PG DNA ends^6^,^7^ and release phosphoglycolate (PG), which is believed to be further hydrolysed by dedicated phosphoglycolate phosphatases (PGPs) in the cytosol. Due to the lack of PGP-deficient model systems, the physiological roles of PG in mammalian organisms have thus far remained elusive.

PGPs are evolutionarily ancient enzymes of the haloacid dehalogenase (HAD)-type superfamily^8^. Although mammalian PGP (also referred to as AUM) is broadly expressed in all investigated tissues and cells^9^, little is currently known about its *in vivo* roles. It has recently been discovered that PGP can operate as a glycerol-3-phosphate (Gro3P) phosphatase, and affect sugar and lipid metabolism in pancreatic β-cells and hepatocytes under metabolic overflow conditions^10^. Nevertheless, the activity of purified, recombinant murine PGP towards PG is at least three orders of magnitude higher than towards Gro3P^11^, prompting us to genetically explore the physiological functions of PGP activity in mice.

Here, we report the unexpected finding that PGP activity is essential for mouse embryonic development. Our results reveal a novel mechanism linking a product of oxidative DNA damage repair to cellular glycerolipid partitioning and cell proliferation.

## Results

### Whole-body PGP inactivation is embryonically lethal

**Figure 1:**
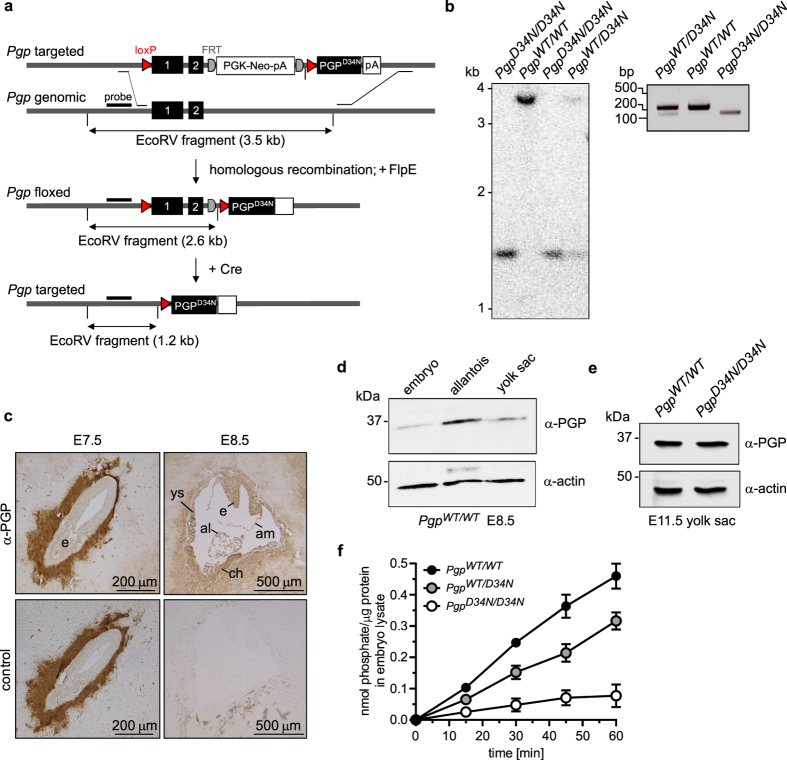
Targeting of a phosphatase-inactive *Pgp* mutant into the murine *Pgp* locus. (**a**) *Pgp* targeting strategy. (**b**) Homologous recombination after breeding *Pgp*^*flx/flx*^ mice with whole-body EIIa-Cre deleter mice, detected by Southern blot analysis (left panel) or by PCR-based genotyping (right panel). (**c**) Immunohistochemical expression analysis of PGP. Uterus buds at E7.5 and E8.5 were cryosectioned and stained with α-PGP antibodies; primary antibodies were omitted in adjacent control sections. ch, chorion; al, allantois; e, embryo; am, amnion; ys, yolk sac. Note that the peri-embryonic signal detected at E7.5 is non-specific. (**d**) Western blot analysis of PGP expression levels. A representative immunoblot of one embryo, three pooled allantoides and one yolk sac at E8.5 is shown. (**e**) Comparison of PGP protein expression in *Pgp*^*WT/WT*^ and *Pgp*^*D34N/D34N*^yolk sacs at E11.5. Blots were reprobed with α-actin antibodies to test for comparable protein loading. (**f**) PGP activity assay in E8.5 embryo lysates. Free phosphate released from exogenous PG was measured using malachite green. Shown are mean values of activity measurements in multiple individual embryos (*Pgp*^*WT/WT*^, *n* = 7; *Pgp*^*WT/D34N*^, *n* = 5; *Pgp*^*D34N/D34N*^, *n* = 5) ± S.E.M. Error bars not seen are hidden by the symbols.

We generated conditionally PGP-inactivated mice by knockin replacement of *Pgp* with its phosphatase-inactive *Pgp*^*D34N*^ mutant in the endogenous locus (Fig. 1a). Southern blot analysis and PCR screening (Fig. 1b) demonstrated homologous recombination after breeding *Pgp*^*flx*/*flx*^ mice with the whole-body Cre deleter strain EIIa-Cre. PGP protein became detectable in embryonic and extraembryonic tissues at embryonic day (E) 8.5 (Fig. 1c,d), and quantitative real-time PCR (qPCR) analysis demonstrated comparable *Pgp* RNA expression levels in E8.5 *Pgp*^*WT*/*WT*^ and *Pgp*^*D34N*/*D34N*^embryos. GAPDH-normalised mean C_t_-values (±S.D.) were 29.75 ± 0.07 for *Pgp*^*WT*/*WT*^ (*n* = 3), and 28.63 ± 0.56 for *Pgp*^*D34N*/*D34N*^ (*n* = 4) embryos. PGP protein levels were also similar in *Pgp*^*WT*/*WT*^ and *Pgp*^*D34N*/*D34N*^ yolk sacs at E11.5 (Fig. 1e). Phosphatase activity against phosphoglycolate (PG) was present in lysates of *Pgp*^*WT*/*WT*^ E8.5 embryos, but close to background values in *Pgp*^*D34N*/*D34N*^ embryos; *Pgp*^*WT*/*D34N*^ embryos had intermediate PG-dephosphorylating activity (Fig. 1f).

Heterozygous PGP-deficient mice were indistinguishable from their wildtype littermates in terms of fertility and growth. Intercrossing *Pgp*^*WT/D34N*^ mice produced offspring, ~64% of which were heterozygotes, and ~36% wildtype. No *Pgp*^*D34N/D34N*^ mice were found at postnatal day 21 (Table 1). The examination of >600 embryos from *Pgp*^*WT/D34N*^ intercrosses at different stages of gestation revealed that genotype ratios were consistent with the expected Mendelian distribution between E8.5 and E11.5, whereas only one homozygous *Pgp* mutant embryo was found at E12.5 (Table 1). Somite pair numbers were comparable at E8.5 in embryos of all genotypes, yet further somitogenesis stagnated in *Pgp*^*D34N*/*D34N*^ embryos (Table 2), indicating that PGP inactivation impaired development beyond E8.5.

### PGP inactivation retards embryonic growth, but does not primarily affect vascular development or placenta formation

**Figure 2:**
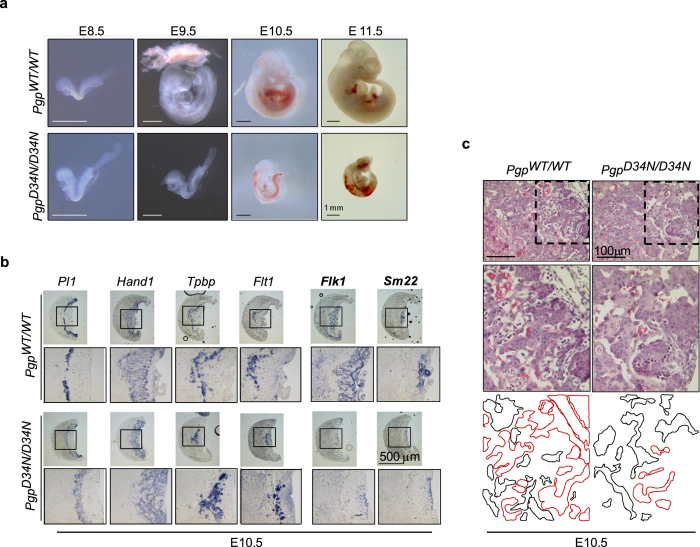
Loss of PGP activity is embryonically lethal in midgestation. (**a**) Comparison of *Pgp*^*D34N/D34N*^ and *Pgp*^*WT/WT*^ embryos from the E8.5 to E11.5 stage of development. (**b**) RNA *in situ* hybridisation of placentae. Markers: *Pl1*, trophoblast giant cells; *Hand1*, trophoblast giant cells, spongiotrophoblasts and parts of labyrinthine layer; *Tpbp* and *Flt1*, spongiotrophoblasts; *Flk1* and *Sm22*, labyrinthine layer. (**c**) Histochemistry of the placental labyrinth. The boxed areas in the upper panels are shown magnified in the middle panels. Lower panel, maternal blood sinuses or embryonic vessels containing immature, nucleated erythrocytes are contoured with black or red lines, respectively. The images are representative of *n* = 8 *Pgp*^*WT/WT*^ and *n* = 11 *Pgp*^*D34N/D34N*^ placentae.

At E9.5, *Pgp*^*D34N*/*D34N*^ embryos resembled E8.5 *Pgp*^*WT/WT*^ embryos in size, and showed a delay in axial rotation (turning), a hallmark of the transition from the E8.5 to E9.5 stage of development (Fig. 2a and Supplementary Table S1). Eight of ten *Pgp*^*WT*/*WT*^ and 22 of 24 *Pgp*^*WT*/*D34N*^ embryos, yet none of eight investigated *Pgp*^*D34N*/*D34N*^ embryos had concluded turning by E9.5. At E10.5, ~80% of *Pgp*^*D34N*/*D34N*^ embryos were growth-retarded, as judged by a size comparable to E9.5 *Pgp*^*WT*/*WT*^ embryos. In addition, beating hearts could only be detected in a fraction of *Pgp*^*D34N*/*D34N*^ embryos, and some embryos additionally had cranial, dorsal and abdominal bleedings; these characteristics were aggravated from E10.5 to E11.5 (Supplementary Table S1 and Fig. 2a).

Between ~E8.5 and E12.5, the growth of murine embryos is nearly exponential^12^, and becomes dependent on the formation of an embryonic cardiovascular system and on the development of the placenta for appropriate oxygen and nutrient supply^13^,^14^. Vascular and placental defects are therefore common causes of embryonic lethality at this developmental stage. However, except for a general developmental delay, the vascularisation of the embryo proper and of extraembryonic *Pgp*^*D34N*/*D34N*^ tissues appeared normal (Supplementary Fig. S1 and Supplementary Text).

Placentae of *Pgp*^*D34N*/*D34N*^ embryos were typically smaller than those of their wildtype littermates, paralleling the growth retardation of *Pgp*^*D34N*/*D34N*^ embryos. The comparison of similarly-sized, E10.5 *Pgp*^*D34N*/*D34N*^ and E10.5 *Pgp*^*WT*/*WT*^ placentae by *in situ* hybridisation demonstrated that the outer and middle placental layers containing *Pl1*- or *Hand1*-positive trophoblast giant cells and *Tbpb*- or *Flt1*-positive spongiotrophoblast cells were properly developed. In contrast, vessel formation in the labyrinthine layer of *Pgp*^*D34N*/*D34N*^ placentae was impaired, as indicated by a strong reduction of *Flk1*- and *Sm22*-positive structures (Fig. 2b). Analysis of histochemical specimens showed that *Pgp* inactivation resulted in a decrease of the embryonic blood vessel density in the placental labyrinth (Fig. 2c); the ratios between embryonic/maternal labyrinthine vessel areas at E10.5 were 13/87% in *Pgp*^*D34N/D34N*^ placentae (*n* = 6), compared to 44/56% (*n* = 4) in *Pgp*^*WT/WT*^ placentae. Nevertheless, since the developmental delay of PGP-inactivated embryos became manifest before the onset of placenta vascularisation (see Fig. 2a), a primary role of impaired labyrinth formation for embryonic growth retardation appears unlikely. In agreement with this conclusion, the genetic ablation of PGP-activity specifically in haematopoietic and endothelial cells did not result in embryonic lethality. *Pgp*^*flx*/*flx*^; *Tie2-Cre*^*+*/*−*^ mice were born at the expected Mendelian ratios, showed no haemorrhages, and were comparable to wildtype mice in terms of viability, growth and fertility. PGP inactivation in these mice was confirmed by analysing phosphatase activity in red blood cell lysates^15^ (Supplementary Figs S2, S3 and Supplementary Methods). Thus, rather than precluding the establishment of embryonic-maternal connections or vascularisation processes *per se*^13^, it appears that loss of PGP activity primarily caused a severe growth defect.

### PGP inactivation blocks cell proliferation under normoxic conditions

**Figure 3:**
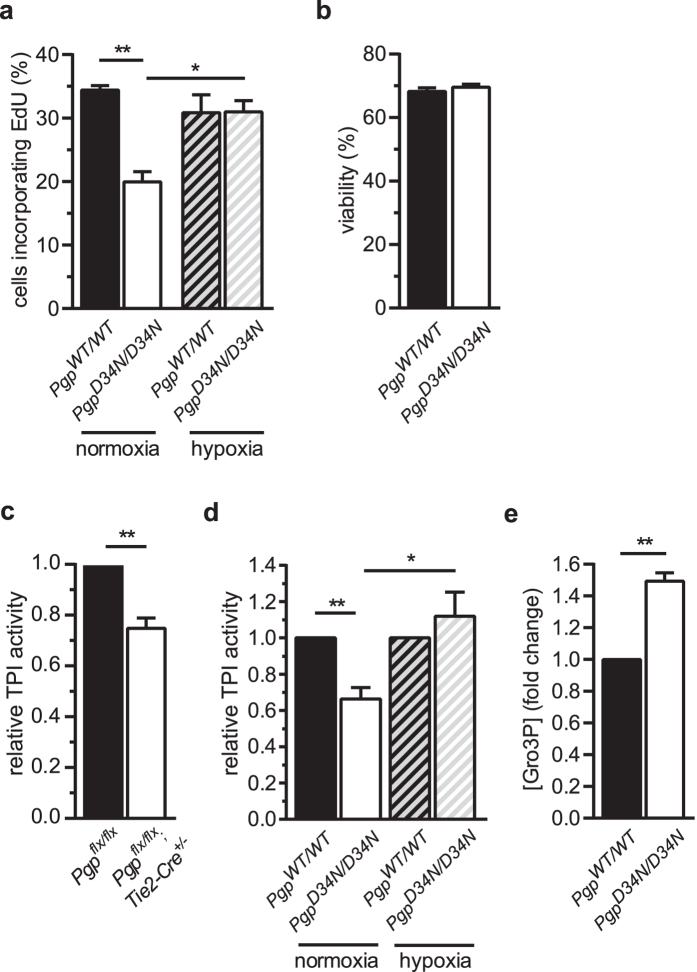
PGP inactivation blocks proliferation, attenuates TPI activity, and increases Gro3P levels. (**a**) Effect of normoxia (~20% O_2_) and hypoxia (~1% O_2_) on the proliferation of MEFs derived from E8.5 embryo explant cultures. (**b**) Cytotoxicity in MEFs derived from E8.5 embryo explant cultures was assessed by LDH release from cells into the medium, and cell viability was expressed as percentage of the total LDH activity present in medium and cells, which was determined after cell lysis. TPI activity in (**c**) erythrocytes and (**d**) E8.5 embryos. TPI activity was normalised to control erythrocytes or to control embryos, respectively. (**e**) Gro3P levels in E8.5 embryos. All results are mean values ± S.E.M., with *n* = 3 independent experiments per condition and genotype. *p < 0.05; **p < 0.01.

Concomitant with the development of the embryonic cardiovascular system and placenta formation, embryos transition from an environment that is physiologically low in oxygen (<2% O_2_) to conditions that approach normoxia once maternal/foetal gaseous exchange has been established^16^. To investigate the potential impact of oxygen on PGP-dependent cell proliferation, we generated mouse embryonic fibroblasts (MEFs). While MEFs could easily be established from E8.5 *Pgp*^*WT*/*WT*^ embryos by dissociation, cells derived from E8.5 *Pgp*^*D34N*/*D34N*^ embryos did not grow under these conditions. In contrast, undissociated E8.5 *Pgp*^*D34N*/*D34N*^ embryos could readily be kept in culture for up to seven days, and beating hearts and cellular outgrowths were observed in all explants. Still, fewer cells grew out from *Pgp*^*D34N*/*D34N*^ than from *Pgp*^*WT*/*WT*^ embryos. These observations suggested that hypoxic conditions –which likely prevail in the interior of undissociated embryo explants– may sustain the viability and proliferation of PGP-deficient cells. Indeed, when PGP-deficient MEFs were obtained by dissociation of embryo explant cultures, their proliferation under normoxic (~20% O_2_) conditions was impaired compared to wildtype MEFs. Under hypoxic (~1% O_2_) conditions however, the proliferation of PGP-inactivated MEFs was completely normalised (Fig. 3a). We did neither detect a decrease in cell viability in *Pgp*^*D34N*/*D34N*^ compared to *Pgp*^*WT*/*WT*^ MEFs derived from E8.5 embryo outgrowths (Fig. 3b), nor an increase in apoptotic cells (*Pgp*^*WT*/*WT*^: 14.6 ± 2.8%, *Pgp*^*D34N*/*D34N*^: 15.3 ± 3.7% TUNEL-positive MEFs; *n* = 2 per genotype). In addition, the analysis of whole mount TUNEL stainings of E8.5 *Pgp*^*WT/WT*^ and *Pgp*^*D34N/D34N*^ embryos did not show a noticeable induction of apoptosis upon PGP-inactivation (Supplementary Fig. S4.) We conclude that oxygen caused a proliferation defect in PGP-inactivated cells.

### PGP inactivation attenuates triosephosphate isomerase activity

PG is generated during the repair of oxidative DNA lesions^3^, and DNA repair genes capable of trimming 3′-PG ends are expressed in E8.5–E11.5 mouse embryos^17^. Midgestational mouse embryos employ both glycolytic and oxidative metabolism^18^,^19^. The latter causes oxidative DNA damage and will thus produce PG, although the amounts of PG that are generated during this period of embryonic development are unknown. Our attempts to measure endogenous PG levels in embryos by gas chromatography coupled to mass spectrometry were unsuccessful due to insufficient sensitivities of the available methods. We therefore analysed whether PGP inactivation impacted PG-dependent functions. *In vitro*, PG has been characterised as a transition state analogue and reversible inhibitor of triosephosphate isomerase (TPI)^20^,^21^, a key glycolytic enzyme that controls a major branching point between glucose and lipid metabolism. Because TPI deficiency in humans can manifest as haemolytic anaemia^22^, we first measured TPI activity in red blood cells isolated from *Pgp*^*flx*/*flx*^*; Tie2-Cre*^*+*/*−*^ mice. PGP inactivation indeed caused a reduction of TPI activity by ~25%, compared to erythrocyte lysates obtained from *Pgp*^*flx*/*flx*^ control mice (Fig. 3c). TPI activity was also inhibited by ~34% in lysates of E8.5 *Pgp*^*D34N*/*D34N*^ embryo explants compared to their wildtype counterparts, consistent with an elevation of PG levels upon PGP inactivation, and this effect was abolished under hypoxic conditions (Fig. 3d). A similar extent of TPI inhibition has been observed in a *S. cerevisiae* strain expressing human wildtype TPI in a yeast *Tpi1*-deficient background, and is associated with increased oxidant resistance by re-routing the carbohydrate flux to the pentose phosphate pathway^23^,^24^.

TPI isomerises the fructose 1,6-bisphosphate cleavage products dihydroxyacetone phosphate (DHAP) and glyceraldehyde 3′-phosphate (GADP). TPI inhibition increases the levels of DHAP, whose formation is thermodynamically favoured^24^, and DHAP can be reversibly reduced to the glycerolipid backbone precursor glycerol-3-phosphate (Gro3P) by glycerol-3-phosphate dehydrogenase. Indeed, we also found higher concentrations of Gro3P in lysates of E8.5 *Pgp*^*D34N/D34N*^ embryos (Fig. 3e). These results are in line with increased levels of DHAP-derived Gro3P upon PG-mediated TPI inhibition, and may additionally result from a direct Gro3P-activity of PGP^10^,^11^.

### PGP inactivation causes triglyceride build-up and phosphatidylcholine depletion

**Figure 4:**
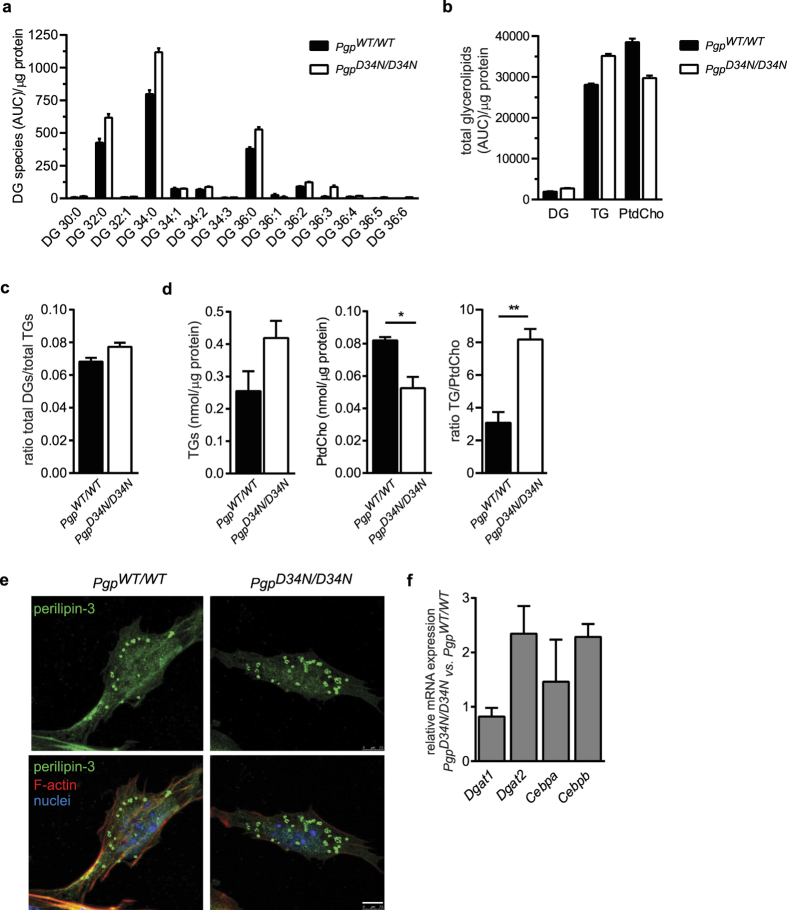
PGP inactivation affects glycerolipid partitioning. (**a**) LC/MS-based determination of diacylglyceride (DG) species in total lipid extracts of 10 pooled E8.5 embryos per genotype. Mean peak area (AUC) values of triplicate determinations ±S.D. are shown. (**b**) LC/MS-based determination of total glycerolipid species (see **a**). Note that the ionisation efficiency of PtdCho species is higher than of TGs (see absolute values in **d**). (**c**) Ratios of total DG/TG species, derived from the measurements shown in (**b**). (**d**) Enzymatic quantification of the TG and PtdCho content and of TG/PtdCho ratios present in E8.5 embryos. Results represent mean values ±S.E.M. of *n* = 3 embryos per genotype. (**e**) Representative lipid droplet stainings in MEFs derived from two independent E8.5 embryo explant cultures per genotype. Lipid droplets were stained with perilipin-3 (green, upper panels), F-actin with phalloidin (red), and nuclei with DAPI (blue). The bottom panels show the merged images. Confocal images are shown. Scale bar, 7.5 μm. (**f**) qPCR analysis of the indicated transcripts in *Pgp*^*WT/WT*^ and *Pgp*^*D34N/D34N*^ E8.5 embryos. Data are mean values ± S.E.M. of *n* = 4 embryos per genotype. *p < 0.05; **p < 0.01.

Because Gro3P provides the carbohydrate backbone of glycerolipids, we next screened the lipid composition of E8.5 *Pgp*^*WT/WT*^ and *Pgp*^*D34N/D34N*^ embryo extracts using high resolution liquid chromatography/mass spectrometry (LC/MS). Several compounds were increased in *Pgp*^*D34N/D34N*^ compared to *Pgp*^*WT/WT*^ embryos. The two statistically most relevantly up-regulated lipids were identified as diacylglycerol (DG) species (Supplementary Fig. S5). In an independent experiment, we profiled glycerolipids in total lipid extracts of pooled E8.5 embryos, using optimised ionisation of DGs. Consistent with the first experiment, we found an increase of DGs in *Pgp*^*D34N/D34N*^ embryos (Fig. 4a). The three predominant species were DG 32:0, DG 34:0 and DG 36:0, reflecting enhanced *de novo* DG biosynthesis rather than increased triglyceride lipolysis as a source of DGs. We also found elevated triglycerides (TGs), yet reduced phosphatidylcholine (PtdCho) levels (Fig. 4b and Supplementary Fig. S6). Due to the increase in TG content that paralleled elevated DG levels, DG/TG ratios only increased by 13% in *Pgp*^*D34N/D34N*^ compared to *Pgp*^*WT/WT*^ embryos (Fig. 4c). An increased TG- and a diminished cellular PtdCho content was also found in an independent set of experiments, employing enzymatic quantification of glycerolipids; the ratio between TGs and PtdCho was ~2.7-fold higher in E8.5 *Pgp*^*D34N/D34N*^ than in *Pgp*^*WT/WT*^ embryos (Fig. 4d). To further confirm cellular triglyceride enrichment, we stained MEFs derived from E8.5 embryo explants for lipid droplets^25^. We found that 22% of *Pgp*^*WT/WT*^ MEFs (165 of 750 analysed cells; *n* = 2) stained positive for the lipid droplet marker perilipin-3, with an average of 35.8 lipid droplets per cell. In contrast, 26.4% of *Pgp*^*D34N/D34N*^ MEFs (132 of 500 analysed cells, *n* = 2) contained lipid droplets, with an average of 47.8 lipid droplets per cell (Fig. 4e). We conclude that PGP inactivation caused cellular triglyceride accumulation and a dysbalance of glycerolipid partitioning between PtdCho and TG pools.

PtdCho formation requires DGs, which are also the substrates of acyl-CoA:diacylglycerol acyltransferases-1 and -2 (DGAT1/2) in the final and only committed step of TG biosynthesis^26^. In addition to the determination of DGAT activity by DG substrate availability, *Dgat* mRNA expression is known to increase during adipogenic differentiation^27^,^28^. By qPCR, we found that *Dgat2* RNA expression levels were more than doubled in E8.5 PGP-inactivated embryos compared to their wildtype counterparts, whereas *Dgat1* expression was unchanged (Fig. 4f). Furthermore, the expression of genes encoding CCAAT/enhancer-binding proteins-α and -β (*Cebpa* and *Cebpb*), were increased in E8.5 *Pgp*^*D34N/D34N*^ embryos, whereas the peroxisome proliferator-activated receptor-γ2 (*Pparg2*) was still undetectable at this stage of embryonic development. Hence, *Pgp* inactivation induces the expression of *Cebpb*, a very early driver of adipogenesis, which is known to trigger the transactivation of the master adipogenic transcription factor *Cebpa*^29^ and of *Dgat2*^30^.

### PGP controls TPI activity, glycerolipid partitioning and cell proliferation in response to oxidative DNA damage repair

**Figure 5:**
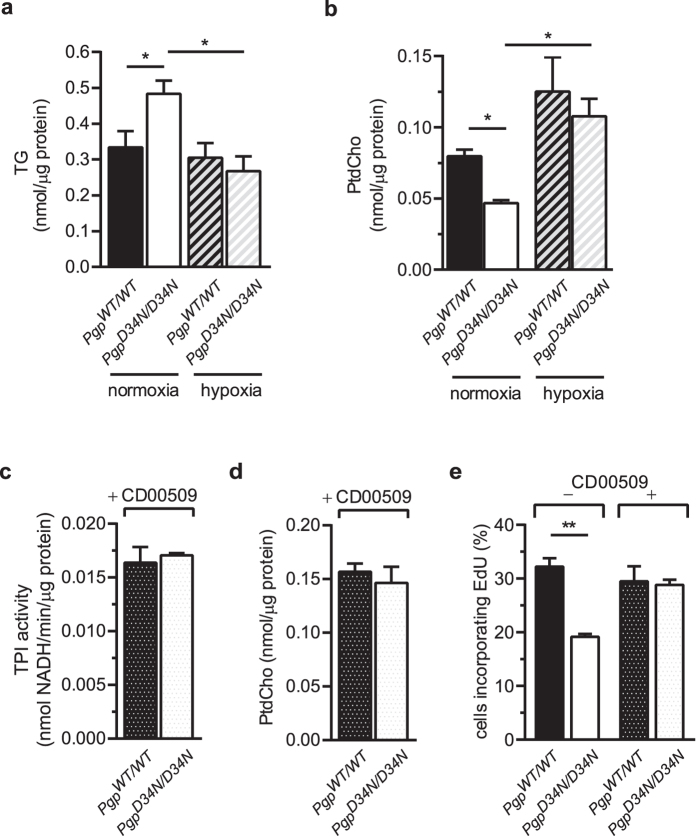
Altered glycerolipid partitioning induced by PGP inactivation is dependent on oxidative DNA damage repair. MEFs derived from E8.5 embryo explant cultures were subjected to normoxia (~20% O_2_) or hypoxia (~1% O_2_), and (**a**) TG and (**b**) PtdCho levels were determined. (**c**) Effect of the TDP1-inhibitor CD00509 (10 μM) on TPI activity, (**d**) PtdCho levels, and (**e**) cell proliferation. In all cases, mean values ± S.E.M. of MEFs derived from *n* = 3 E8.5 embryos per condition and genotype are shown. *p < 0.05; **p < 0.01.

Increased TG and diminished PtdCho levels were also measured in MEFs derived from E8.5 embryo explant cultures. Strikingly, when PGP-inactivated MEFs were kept under hypoxic conditions (*i.e.*, under settings that restored cell proliferation to wildtype levels, see Fig. 3a), TG and PtdCho levels shifted to values comparable to their *Pgp*^*WT/WT*^ counterparts (Fig. 5a,b).

Since PG is released from oxidatively damaged DNA termini bearing 3′-phosphoglycolate ends, which can be repaired by tyrosyl DNA phosphodiesterase-1 (TDP1)^2^,^3^,^6^,^7^,^31^, we examined the impact of TDP1 inhibition. Figure 5c shows that the TDP1 inhibitor CD00509^32^ effectively restored the attenuated TPI activity of PGP-inactivated MEFs to wildtype levels. TDP1 inhibition also normalised PtdCho levels (Fig. 5d), and corrected the reduced cell proliferation of *Pgp*^*D34N/D34N*^ MEFs (Fig. 5e). These results argue that in the absence of PGP activity, TDP1-dependent PG generation impacts cellular TPI activity, and that this is associated with PtdCho depletion and stalled cell proliferation. We conclude that PGP activity safeguards cell proliferation in response to oxidative DNA damage repair.

## Discussion

Here we show that PGP is essential for mouse embryonic growth and development, and reveal a mechanistic link between oxidative DNA damage repair, cellular glycerolipid partitioning and cell proliferation. The growth delay and subsequent demise of PGP-mutant embryos coincided with the gradual increase of oxygen supply to the embryo *via* the developing cardiovascular system. No PGP-inactivated embryos were found beyond the commencement of placenta vascularisation, when normoxic conditions become predominant in the embryo^16^. Although the cardiovascular system and the placenta transport oxygen, these structures initially also develop under hypoxic conditions, which could explain why they were elaborated –albeit in a delayed fashion– in the absence of PGP activity at all.

Under physiological conditions, up to 1–2% of metabolised oxygen is converted to superoxide that can cause oxidative DNA damage^33^. Oxidative stress-induced DNA damage represents a major fraction of the ~10^5^ DNA lesions that occur daily per mammalian cell^1^,^3^, and about 25% of these are strand breaks bearing PG-terminated 3′-overhangs, from which PG is released^34^. Thus, it is plausible that PGP inactivation became phenotypically manifest after E8.5 due to the oxygen-dependent generation of 3′-PG termini commencing at this developmental stage. In support of this hypothesis, pharmacological TDP1 inhibition fully reversed the cell proliferation defect caused by PGP-inactivation under normoxic conditions. Nevertheless, we cannot exclude the possibility that additional factors are regulated in a PGP- and oxygen-dependent manner, and contribute to the observed effects.

PG concentrations in wildtype, postmitotic, PGP-proficient rat hepatocytes have been calculated to <0.04 nmol/mg protein, or <20 μM 10, a concentration that is approximately 10-fold below the *K*_m_ of PGP for PG^11^.Intracellular PG concentrations may physiologically be maintained at low levels by PGP to safeguard the activity of PG-sensitive enzymes such as TPI^35^. Although PG has been used as a TPI transition state inhibitor in enzymatic and structural studies for decades^36^, to our knowledge, this study is the first demonstration that TPI activity is sensitive to PGP inactivation in a mammalian organism.

In *Pgp*^*D34N/D34N*^ embryos or fibroblasts, TPI activity was reduced by ~34%; similar to a *S. cerevisiae* strain expressing human wildtype TPI in a yeast *Tpi1*-deficient background. This yeast strain was viable and showed increased oxidant resistance^24^. Heterozygous *Tpi*-deficient mice are also viable, while homozygote *Tpi* null alleles are embryonically lethal^37^. Moreover, human TPI deficiency is a very rare and lethal genetic disorder, with <100 individuals diagnosed worldwide since the discovery of the syndrome. Yet, subjects exhibiting about 50% of the normal TPI activity are quite common, with allele frequencies of ~0.002 in the Caucasian and ~0.02 in the African American population, and these individuals are clinically unaffected (reviewed in ref. 38). Consequently, the moderate inhibition of TPI activity alone is unlikely to account for the reduced cell proliferation and embryonic lethality caused by PGP inactivation. Because PG is a well-established inhibitor of TPI *in vitro*, we have analysed cellular TPI activity as a readout for PG-dependent effects in the current study. It has to be taken into account that TPI is a highly efficient, catalytically ‘perfect’ enzyme, and since the PG accumulation in PGP-inactivated embryos appears to be sufficient to reduce TPI activity, it can be expected that many other enzymes in parallel will be affected by the accumulation of this small and polar metabolite. Thus, altered activities of multiple PG-sensitive enzymes together may cause the phenotype. The identification of these targets will be an important aim of future studies. In addition, whereas impaired TPI activity in yeast was associated with a ~40% decrease in Gro3P levels^24^, Gro3P levels were increased by ~50% upon PGP inactivation (this work). Hence, we postulate that the dual phosphatase activity of PGP towards PG and Gro3P 10,11 accounts for the observed phenotypes.

In line with an increased availability of Gro3P as an activated carbohydrate backbone for lipogenesis, the PGP-dependent developmental arrest and the proliferation block of embryonic fibroblasts were associated with an elevated cellular TG content. Increased glycerolipid content was restored to normal by TDP1 inhibition, and was thus dependent on PG generation and the impaired PG dephosphorylation in *Pgp*^*D34N/D34N*^ cells. Hence, PG-induced TPI inhibition shifts the triosephosphate equilibrium towards DHAP, which can be reduced to Gro3P, and carbohydrate flux towards *de novo* DG biosynthesis may be favoured by impaired Gro3P dephosphorylation. Facilitated by increased DGAT2 expression, and thus at the expense of the cellular PtdCho content, these saturated, potentially toxic DG species may then preferentially be incorporated into TGs in a cytoprotective response reaction^39^.

PtdCho is a major membrane phospholipid, and genetic deletions of key enzymes in PtdCho biosynthesis result in embryonic lethality in mice^40^,^41^. In addition, the conditional ablation of the rate-limiting enzyme of the PtdCho-producing CDP-choline pathway in a mammalian cell line reduces cellular PtdCho content by ~50% 42, prompting a proliferation block and finally apoptosis^43^. Because of the almost exponential growth of midgestational embryos, and because adequate PtdCho levels are indispensable for membrane biogenesis, growth and proliferation^44^, the ~40% diminished PtdCho content of PGP-inactivated embryos and cells is likely a major contributor to the observed reduction in cell proliferation. Nevertheless, because membrane biogenesis and PtdCho synthesis are regulated in a cell cycle-dependent manner, and PtdCho biosynthesis is elevated in the G1/S-phase^45^,^46^, it is also possible that changes in cell cycle progression contribute to the observed phenotype, and that such cell cycle effects are a consequence of elevated DG and TG levels. Our preliminary analysis of 84 genes involved in cell cycle regulation using a qPCR-based cell cycle array indicated a decrease in *Mki67* (Ki67) and *Ccna2* (cyclin A2) expression in *Pgp*^*D34N*/*D34N*^ compared to *Pgp*^*WT*/*WT*^ E8.5 embryos. In lung cancer cells, it has been shown that the DG-sensitive PKC isoform PKCδ can inhibit cyclin A promoter activity by inducing the expression of the cyclin dependent kinase inhibitor p21^47^. However, we did not observe altered p21 (*Cdkn1*) gene expression levels in *Pgp*^*D34N/D34N*^ embryos. Further mechanistic studies using suitable model cells will be required to elucidate potential links between PGP-dependent glycerolipid partitioning, elevated DG and TG levels, and changes in cell cycle regulation.

The potential involvement of C/EBPβ (and the subsequent induction of C/EBPα expression) in the embryonic phenotype needs further clarification. In addition, it remains to be resolved how the loss of PGP activity can feed back on C/EBPβ expression and activity. One conceivable scenario is that PGP inactivation affects ROS signalling to C/EBPβ. Transient ROS production *via* activation of the NADPH oxidase (NOX) complex is known to be an important cue for the differentiation of various cell types, including preadipocytes^48^. C/EBPβ is critical for embryonic development in the process of decidualisation (*i.e.*, the transformation of fibroblast-like uterine stromal cells to secretory decidual cells)^49^, and depending on the severity of the phenotype, the manifestation of inadequate decidualisation can range from embryo implantation failure to impaired placenta formation and foetal growth restriction. In addition, it has been shown that NOX4-dependent ROS signalling enhances C/EBPβ activity during decidualisation^50^. Hence, altered oxidative stress responses in the absence of PGP activity may also affect ROS signalling to C/EBPβ.

Phosphatases of the haloacid dehalogenase superfamily are enzymes with characteristically moderate catalytic efficiencies^51^, and this feature also applies to PGP^11^. Our work suggests that it may be this moderate PGP activity in itself that is biologically advantageous. It is tempting to speculate that elevated PG levels that temporarily exceed the hydrolytic capacity of PGP relay an oxidative DNA damage signal, and induce a cell protective, metabolic adaptation that halts cell proliferation. The reversible inhibition of PGP by oxidation^11^ may trigger similar cellular responses. Future studies will be dedicated to resolving these intriguing regulatory mechanisms.

## Methods

### Materials

Cell culture media and supplements were from PAN Biotech. Unless otherwise specified, all reagents were of the highest available purity and were purchased from Sigma-Aldrich.

### Generation and breeding of PGP-inactivated mice

Floxed *Pgp* mice (*Pgp*^*tm1Goh*^) were generated on a C57Bl/6J background by Ozgene Pty Ltd., Australia. The neomycin resistance cassette was removed by breeding with the whole-body FLPe deleter strain B6.129S4-Gt(ROSA)26Sor<tm1(FLP1)Dym>/RainJ. Whole-body or haematopoietic/endothelial cell-specific PGP inactivation was achieved by breeding with B6.FVB-Tg(EIIa-cre)C5379Lmgd/J (EIIa-Cre) or B6.Cg-Tg(Tek-cre)1Ywa/J (Tie2-Cre) transgenic mice obtained from The Jackson Laboratory. Mouse experiments were approved by the Regierung Unterfranken, and all analyses were carried out in strict accordance with all German and European Union applicable laws and regulations concerning care and use of laboratory animals.

### Genotyping

Genomic DNA was isolated from ear punch biopsies with DNeasy (Qiagen) and digested with EcoRV. Southern blotting was performed according to standard procedures. The probe was generated by PCR using Pfx polymerase (Invitrogen) and labeled with [α-^32^P]dCTP (Hartmann Analytic) employing the DecaPrime II DNA labeling kit (Ambion). Products are 3.5 kb (*Pgp*^*WT*^), 2.6 kb (*Pgp*^*flx*^) and 1.2 kb (*Pgp*^*D34N*^). Genotyping by PCR was performed from yolk sacs or ear-punch biopsies using DreamTaq (Thermo Fisher). The primers (forward: 5′-AATGAGCGTCCCGGAGGC-3′; reverse: 5′-AAACCCAAGCGCCTTAGC-3′) are complementary to the 50 bp intron that is lacking in the knocked-in *Pgp* minigene, and detect the wildtype (212 bp) or targeted (163 bp) allele. Genotyping of *Tie2-Cre* transgenic mice was performed as described by The Jackson Laboratory.

### Quantitative real-time RT-PCR (qPCR)

Total RNA from E8.5 embryos was isolated using the RNeasy Mini Kit. First-strand cDNA was synthesised with the RT^2^ First Strand Kit, and qPCR was performed with the RT^2^ Sybr Green Mastermix (Qiagen), using *Gapdh* as an internal control for normalisation. The primers used were as follows (given as forward and reverse, respectively): *Cebpa* (5′-AAACAACGCAACGTGGAGA-3′, 5′-GCGGTCATTGTCACTGGTC-3′), *Cebpb* (5′-TGATGCAATCCGGATCAA-3′, 5′-CACGTGTGTTGCGTCAGTC-3′), *Dgat1* (5′- TCGTGGTATCCTGAATTGGTG-3′, 5′-AGGTTCTCTAAAAATAACCTTGCATT-3′), *Dgat2* (5′-GGCGCTACTTCCGAGACTAC-3′, 5′-TGGTCAGCAGGTTGTGTGTC-3′), *Gapdh* (5′-TGGCAAAGTGGAGATTGTTC-3′; 5′-CATTATCGGCCTTGACTGTG-3′), *Pgp* (5′-GAGACTCTGCGGGCTCTG-3′; 5′-AAACCCAAGCGCCTTAGC-3′), *Pparg2* (5′-TGCTGTTATGGGTGAAACTCTG-3′, 5′-CTGTGTCAACCATGGTAATTTCTT-3′). Transcript abundance was determined using the ΔΔC_t_ method. Changes in the expression of cell cycle-related genes in E8.5 embryos were monitored using the RT^2^ profiler PCR array mouse cell cycle (Qiagen).

### PGP activity assay

To assess PGP activity in embryos, individual E8.5 embryos were cultured for seven days as described below and lysed in TMN (30 mM triethanolamine, 30 mM NaCl, 5 mM MgCl_2_; pH 7.5) supplemented with 10 μg/mL aprotinin, 10 μg/mL leupeptin, 1 mM pepstatin and 1 mM 4-(2-aminoethyl)benzenesulfonyl fluoride by shearing through a needle. Cell debris was removed by centrifugation (10,000 × *g*, 5 min). Protein concentrations were determined using the Micro BCA Protein Assay (Thermo Scientific). Reactions (10 μg total protein each) were started by the addition of PG (final concentration, 1 mM) in a total volume of 100 μL TMN supplemented with 1 mM DTT. Aliquots (20 μL) were removed at the indicated time points, and reactions were stopped by the addition of 100 μL malachite green solution (Biomol Green; Enzo Life Sciences). Released phosphate was determined by measuring A_620_ and extrapolating the values to a phosphate standard curve.

### In situ hybridisation and histology

Details of RNA *in situ* hybridisations on placentae and sectioned embryos and the sequences of digoxigenin-labeled riboprobes were described previously^52^. For histological analysis, paraffin-embedded tissues sections were cut (2 μm) and stained with haematoxylin and eosin (HE). Morphometric analysis of placenta vascularisation was performed on HE-stained sections. A central area of the labyrinth was analysed, if possible determined by the presence of the umbilical cord. The fractional area of the labyrinthine vasculature occupied by maternal or embryonic vessels was calculated by contouring the respective vessels using ImagePro-Plus software version 7.0 (Media Cybernetics).

### Embryo explant cultures, cell proliferation, hypoxia, cytotoxicity, apoptosis and TDP1 inhibition

E8.5 embryos were explanted on 24-well microtiter plates precoated with 0.1% gelatine and cultured in Dulbecco’s modified Eagle’s medium containing 25 mM glucose and supplemented with 10% (v/v) foetal calf serum (FCS), 100 U/mL penicillin G, 100 μg/mL streptomycin, and 2 mM L-glutamine (complete DMEM). Heart beats were assessed daily. To generate MEFs, explants were trypsinised after seven days. Per condition, 1,000 MEFs were seeded in DMEM/10% FCS in duplicate wells of 96-well microtiter plates precoated with 0.1% gelatine. To investigate the effect of TDP1 inhibition, cells were incubated for 24 h with 10 μM CD00509 (Merck). The effect of hypoxia on cell proliferation was assessed using a hypoxia chamber (Stemcell Technologies), and cells were kept either at ~20% O_2_, 5% CO_2_ or at ~1% O_2_, 5% CO_2_ for 16 h in the presence of 10 μM 5-ethynyl-2′-deoxyuridine (EdU; Click-iT EdU Alexa Fluor 488 imaging kit, Invitrogen) to assay for DNA synthesis. Apoptosis was analysed by TUNEL staining (Roche). Cells were counterstained with DAPI and imaged on a Nikon TE Eclipse epifluorescence microscope equipped with a 4 × objective, and EdU or TUNEL and/or DAPI-labelled cells were analysed using Image Pro software. Approximately 500–800 cells were scored per condition. Apoptosis in whole-mount E8.5 embryos was detected by TUNEL staining, and specimens were analysed using a Zeiss LSM510 META confocal microscope. Cell viability was determined under the conditions of the cell proliferation assay, by measuring lactate dehydrogenase (LDH) release from the cells into the medium using the Pierce LDH Cytotoxicity Assay (Life Technologies). Cytotoxicity was expressed as a percentage of the total LDH activity present in medium and cells, determined after cell lysis.

### Lipidomics

All solvents were LC/MS grade and purchased from Biosolve. Per experiment, ten pooled E8.5 embryos (*Pgp*^*WT*/*WT*^ or *Pgp*^*D34N*/*D34N*^; ~50 μg total protein) were extracted with 600 μL 75% methanol. Samples were homogenised in a ball mill equipped with zirconium oxide grinding balls (MM 301 Mixer Mill; Retsch GmbH). Lipids were extracted using *tert*-butylmethylether (TBME) as described^53^ and analysed by LC/MS as published previously^54^. Processing of chromatograms, peak detection and integration were performed using MassLynx software (version 4.1, Waters). TransOmics software (Waters) was used for data preprocessing for untargeted metabolomics and for marker identification. Glycerolipid profiling was conducted using the in-house developed software RLA-Tool (A.F. and M.J.M., unpublished). Defined lipid species were searched for based on their exact m/z in the low energy function and two integral fragments (fatty acyl side chains) in the high energy function (error tolerance, 3 mDa). The identification was confirmed by the linear coherence between retention time and m/z. The identified species were then assembled into an automated method within QuantLynx embedded in MassLynx for the systematic integration of the identified lipid species in the extracts.

### TPI activity, Gro3P levels, enzymatic PtdCho and TG determination and lipid droplet staining

TPI activity in MEFs derived from E8.5 embryo explant cultures and in red blood cells was measured using the Triose Phosphate Isomerase Activity Kit, and Gro3P levels were determined using the Glycerol-3-Phosphate Assay Kit (both from BioVision). Total PtdCho and TG levels were quantified with the Phosphatidylcholine Assay or the Triglyceride Determination Kit (both from Sigma), according to the manufacturer’s instructions. For the visualisation of lipid droplets, MEFs were fixed for 15 min with 4% paraformaldehyde, permeabilised and blocked with 0.2% saponin/2% bovine serum albumin (BSA) for 1 hr, and immunostained for 1 hr using α-rabbit TIP47/perilipin 3 antibodies (a generous gift of Dr Christoph Thiele) in 0.2% saponin/1% BSA^25^. Primary antibodies were detected with Alexa Fluor 488-labeled goat anti-rabbit secondary antibodies (1:400; Molecular Probes/Thermo Fisher Scientific). Filamentous actin was stained with phalloidin-Alexa Fluor 546 (1:400; Molecular Probes/Thermo Fisher Scientific), and nuclei were visualised with 4′,6-diamidino-2-phenylindol (DAPI). Images were captured using a Zeiss LSM510 META confocal microscope.

### Image quantification and statistical analysis

Western blots were quantified with NIH ImageJ, version 1.45i. For statistical analysis, two-tailed, unpaired Student’s *t*-tests were performed using GraphPad Prism version 6.0.

## Additional Information

**How to cite this article**: Segerer, G. *et al.* An essential developmental function for murine phosphoglycolate phosphatase in safeguarding cell proliferation. *Sci. Rep.* **6**, 35160; doi: 10.1038/srep35160 (2016).

## Supplementary Material

Supplementary Information

## Figures and Tables

**Table 1:** Characterisation of progeny from heterozygous intercrosses.

genotype			
stage	*Pgp*^*WT/WT*^	*Pgp*^*WT/D34N*^	*Pgp*^*D34N/D34N*^
E8.5	138	294	104
E9.5	13	34	12
E10.5	25	49	30
E11.5	16	29	18
E12.5	6	18	1
P21	30	53	0

E, embryonic day; P, postnatal day.

**Table 2:** Embryo staging.

number of somite pairs			
stage	*Pgp*^*WT/WT*^	*Pgp*^*WT/D34N*^	*Pgp*^*D34N/D34N*^
E8.5	4–6	4–6	4–6
	(*n* = 4; n.d. = 2)	(*n* = 12; n.d. = 3)	(*n* = 4; n.d. = 1)
E9.5	18–24	16–23	4–6
	(*n* = 4; n.d. = 2)	(*n* = 9; n.d. = 6)	(*n* = 3; n.d. = 1)

E, embryonic day; *n*, number of scored embryos; n.d., not definable.
