# Supplementary material for: An essential developmental function for murine phosphoglycolate phosphatase in safeguarding cell proliferation
Source: Sci Rep. 2016 Oct 12;6:35160. doi: 10.1038/srep35160 (PMC5059750; doi:10.1038/srep35160)
Supplement: Supplementary Information [file srep35160-s1.pdf]

SUPPLEMENTARY INFORMATION

An essential developmental function for murine phosphoglycolate phosphatase in safeguarding cell proliferation

Gabriela Segerer, Kerstin Hadamek, Matthias Zundler, Agnes Fekete, Annegrit Seifried, Martin J. Mueller, Frank Koentgen, Manfred Gessler, Elisabeth Jeanclos, Antje Gohla

Fig. S1

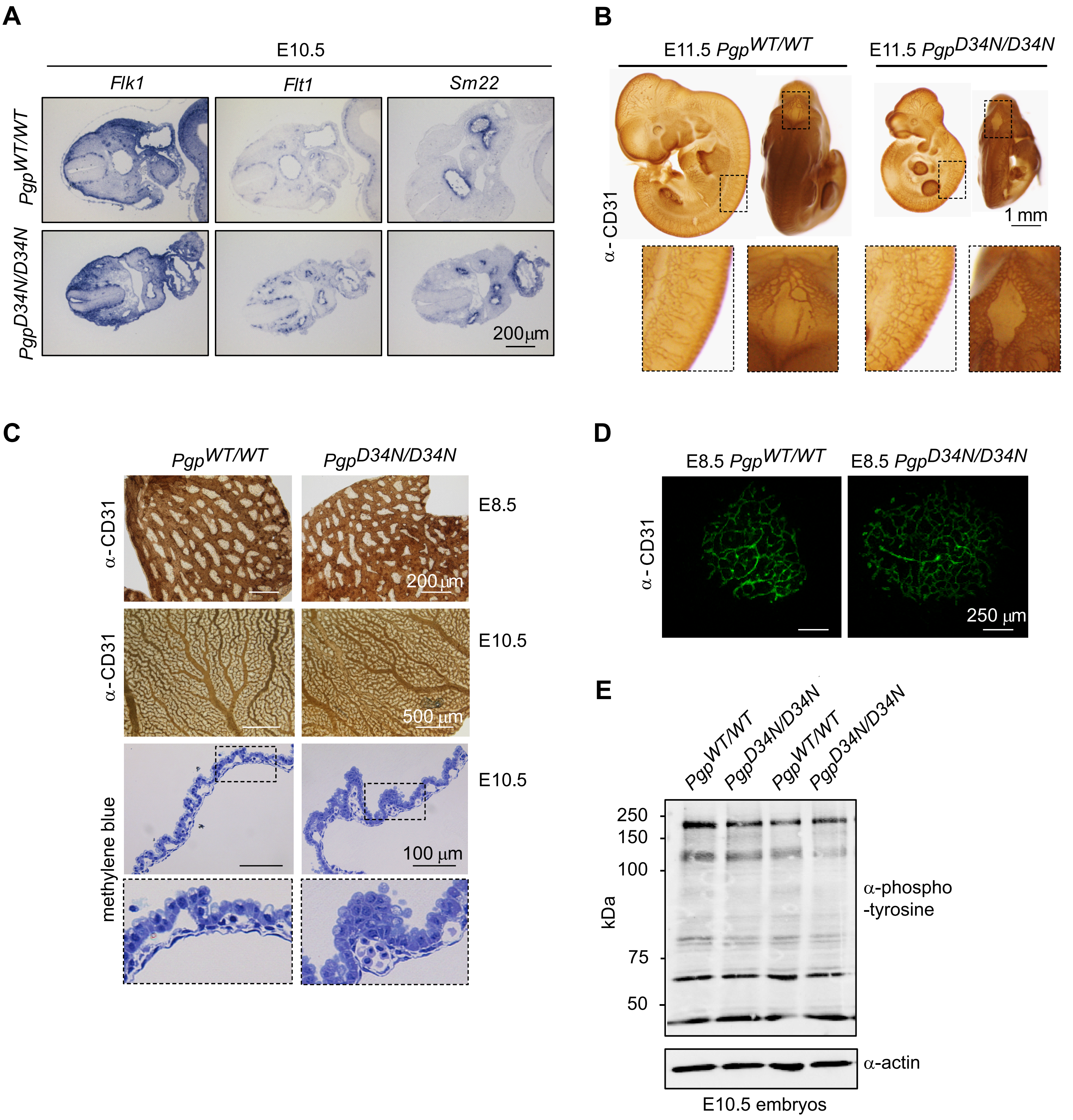
A
E10.5
Flk1
Flt1
Sm22
PgpWT/WT
PgpD34N/D34N
200μm
B
E11.5 PgpWT/WT
E11.5 PgpD34N/D34N
α-CD31
1 mm
C
PgpWT/WT
PgpD34N/D34N
α-CD31
E8.5
200 μm
α-CD31
E10.5
500 μm
methylene blue
E10.5
100 μm
D
E8.5 PgpWT/WT
E8.5 PgpD34N/D34N
α-CD31
250 μm
E
PgpWT/WT
PgpD34N/D34N
PgpWT/WT
PgpD34N/D34N
250
150
100
75
50
kDa
α-phospho
-tyrosine
α-actin
E10.5 embryos

Fig. S2

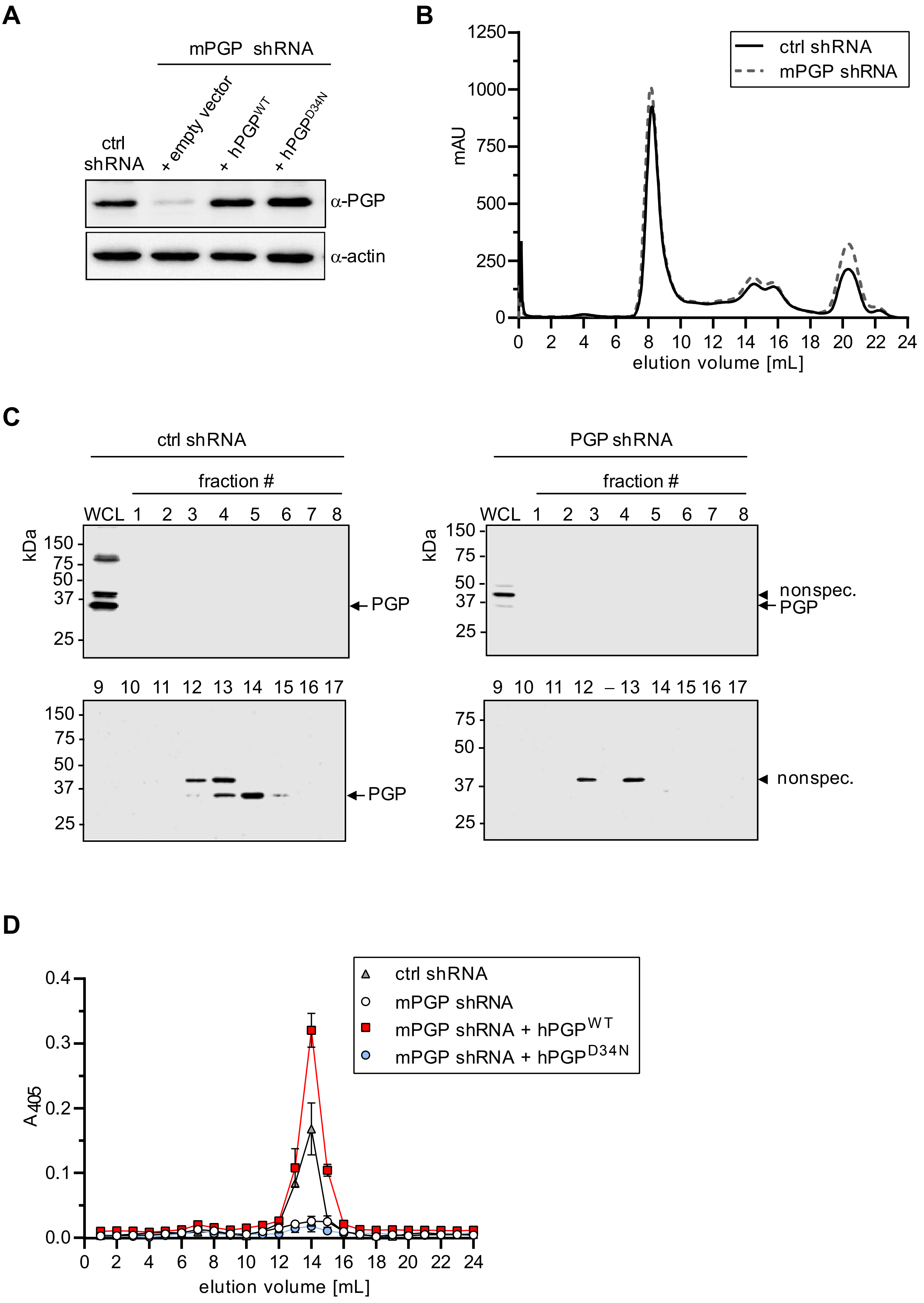

A
mPGP shRNA
ctrl shRNA
+ empty vector
+ hPGP^WT
+ hPGP^D34N
α-PGP
α-actin
B
ctrl shRNA
mPGP shRNA
mAU
1250
1000
750
500
250
0
0 2 4 6 8 10 12 14 16 18 20 22 24
elution volume [mL]
C
ctrl shRNA
fraction #
WCL 1 2 3 4 5 6 7 8
kDa
150
75
50
37
25
PGP
9 10 11 12 13 14 15 16 17
PGP shRNA
nonspec.
PGP
9 10 11 12 – 13 14 15 16 17
nonspec.
D
ctrl shRNA
mPGP shRNA
mPGP shRNA + hPGP^WT
mPGP shRNA + hPGP^D34N
A405
0.4
0.3
0.2
0.1
0.0
0 2 4 6 8 10 12 14 16 18 20 22 24
elution volume [mL]

Fig. S3

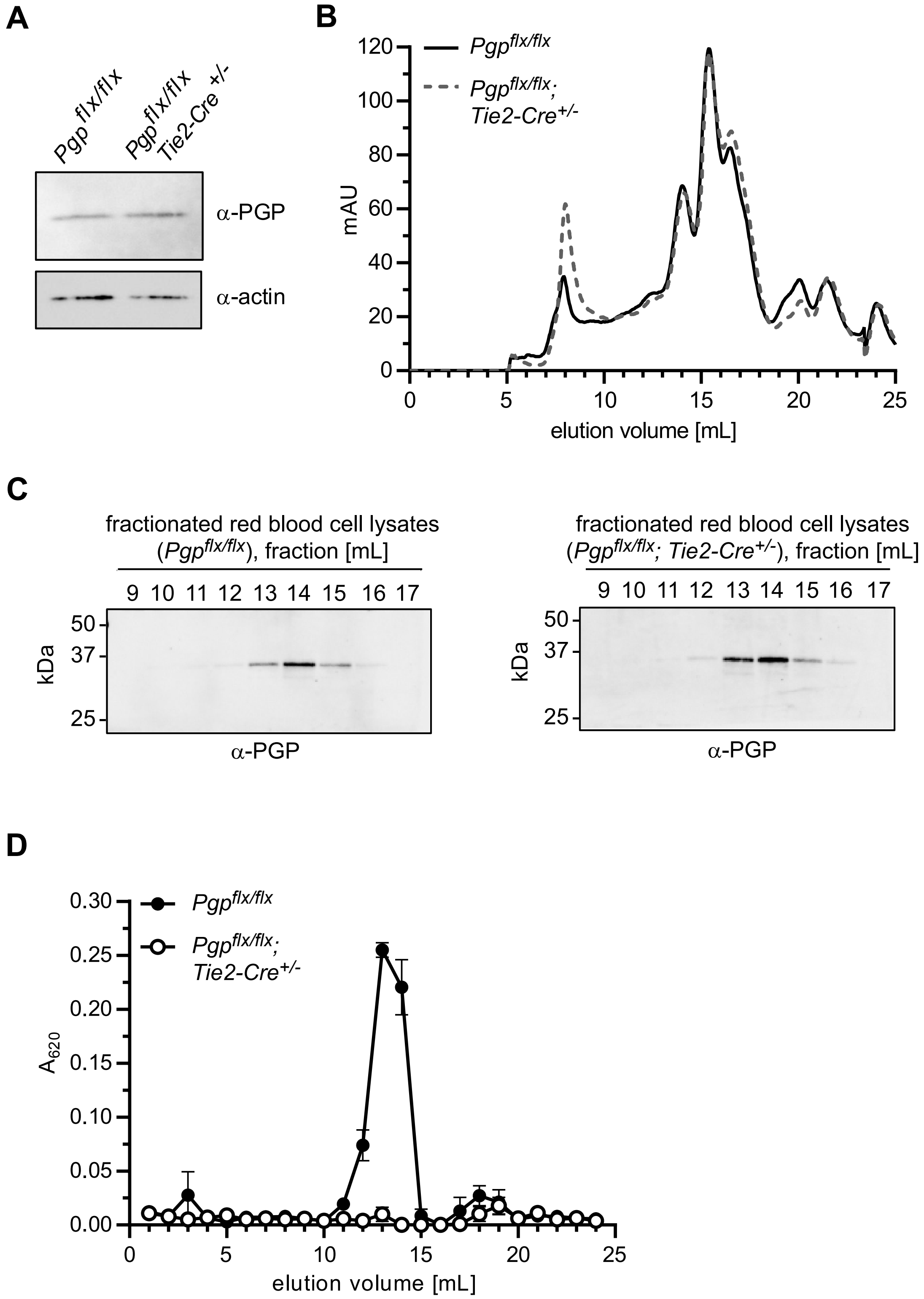
A
Pgp flx/flx
Pgp flx/flx Tie2-Cre +/-
α-PGP
α-actin
B
Pgp flx/flx
Pgp flx/flx; Tie2-Cre+/-
mAU
elution volume [mL]
C
fractionated red blood cell lysates (Pgp flx/flx), fraction [mL]
fractionated red blood cell lysates (Pgp flx/flx; Tie2-Cre+/-), fraction [mL]
9 10 11 12 13 14 15 16 17
kDa
50
37
25
α-PGP
D
Pgp flx/flx
Pgp flx/flx; Tie2-Cre+/-
A620
elution volume [mL]

Fig. S4

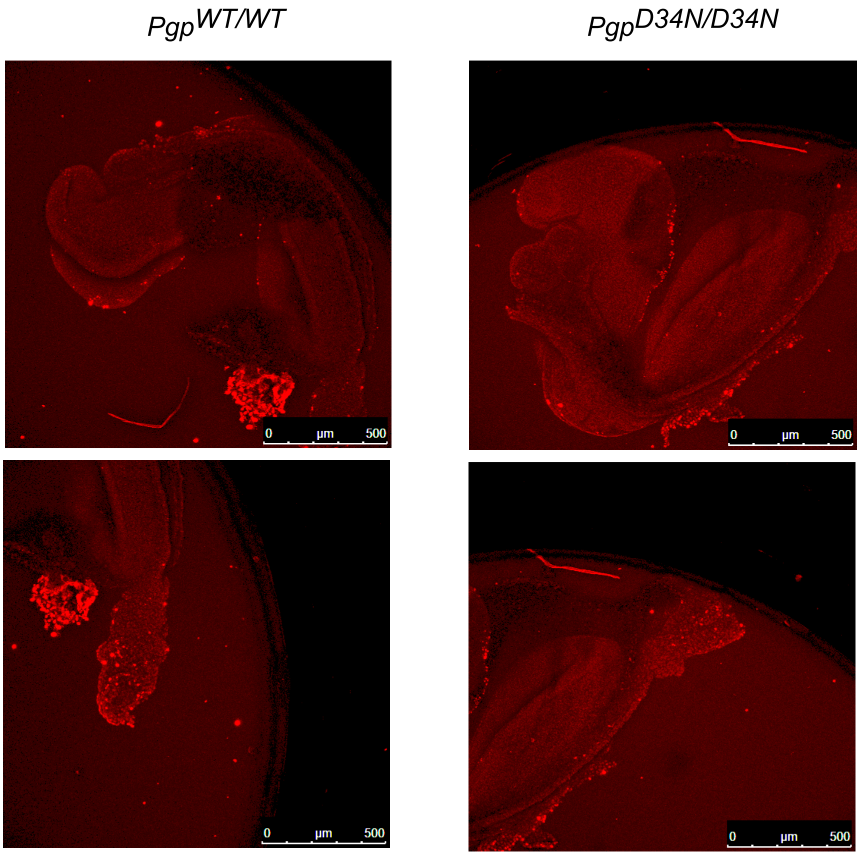

Fig. S5

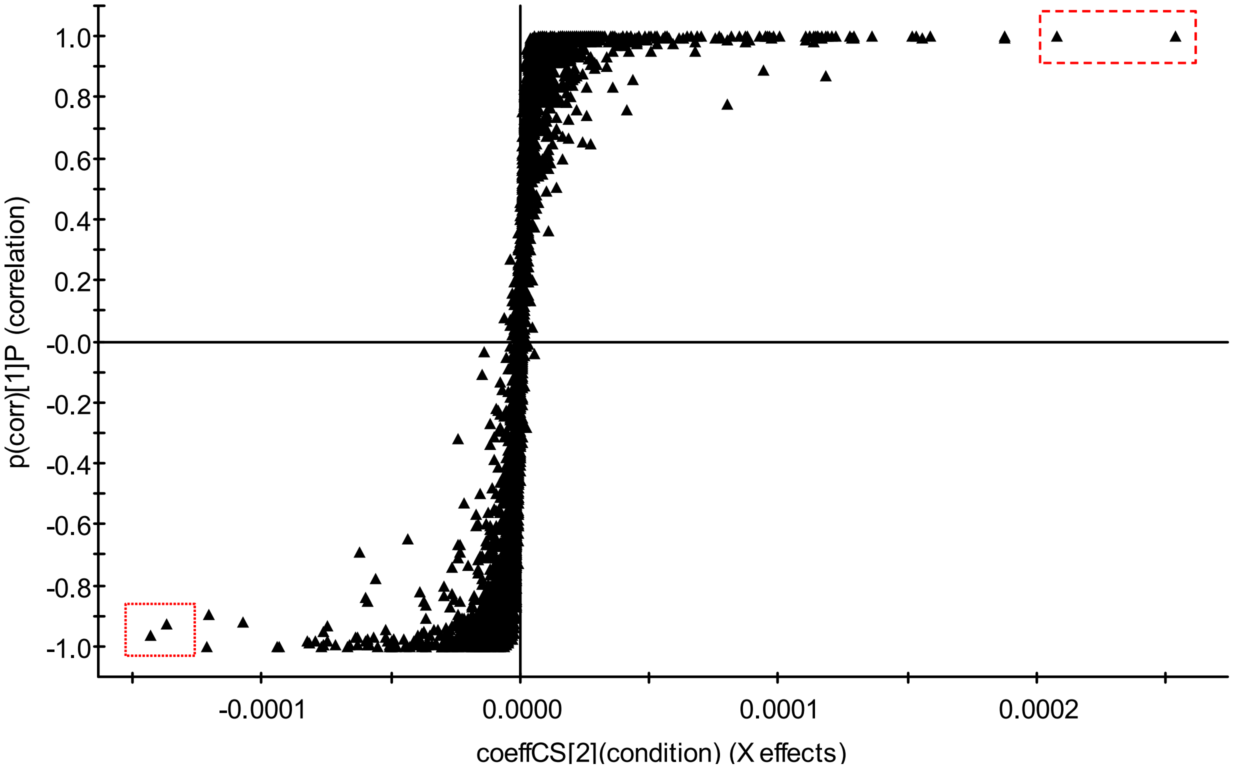
1.0
0.8
0.6
0.4
0.2
-0.0
-0.2
-0.4
-0.6
-0.8
-1.0
p(corr)[1]P (correlation)
-0.0001
0.0000
0.0001
0.0002
coeffCS[2](condition) (X effects)

Fig. S6

A

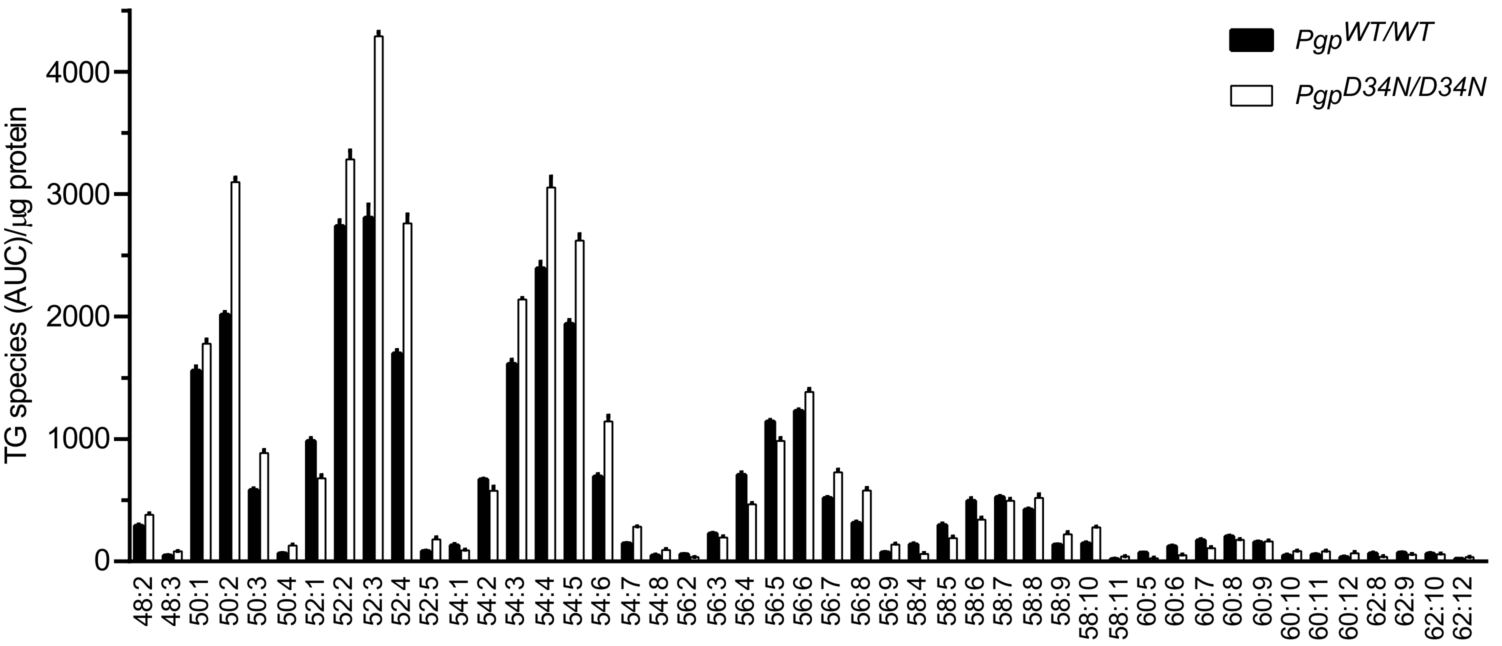

TG species (AUC)/µg protein
4000
3000
2000
1000
0
48:2
48:3
50:1
50:2
50:3
50:4
52:1
52:2
52:3
52:4
52:5
54:1
54:2
54:3
54:4
54:5
54:6
54:7
54:8
56:2
56:3
56:4
56:5
56:6
56:7
56:8
56:9
58:4
58:5
58:6
58:7
58:8
58:9
58:10
58:11
60:5
60:6
60:7
60:8
60:9
60:10
60:11
60:12
62:8
62:9
62:10
62:12
Pgp WT/WT
Pgp D34N/D34N

B

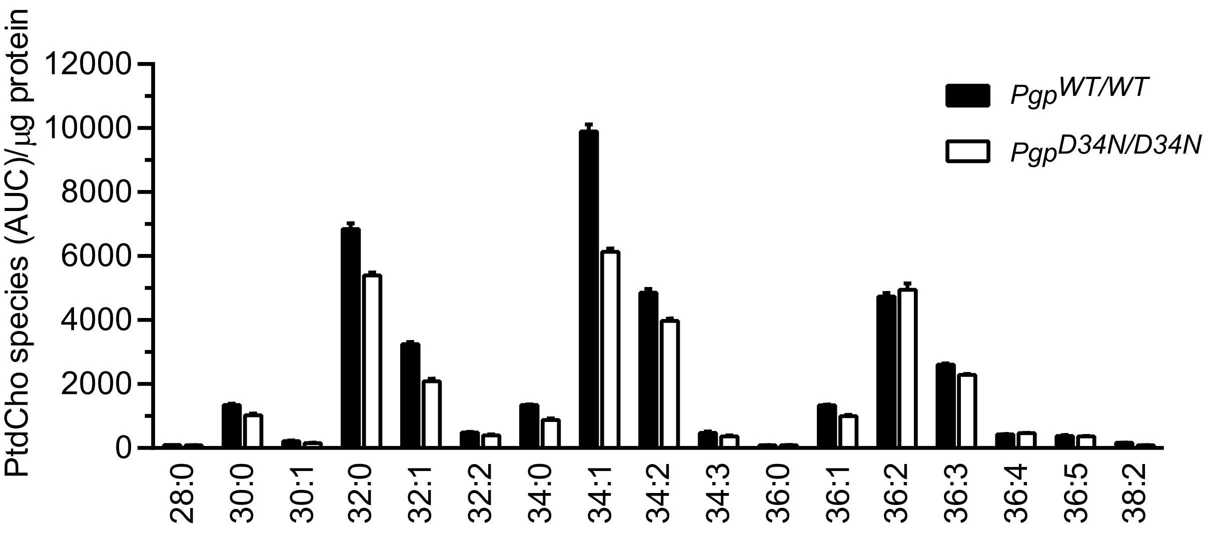

PtdCho species (AUC)/µg protein
12000
10000
8000
6000
4000
2000
0
28:0
30:0
30:1
32:0
32:1
32:2
34:0
34:1
34:2
34:3
36:0
36:1
36:2
36:3
36:4
36:5
38:2
Pgp WT/WT
Pgp D34N/D34N

SUPPLEMENTARY METHODS

Cell culture, red blood cell isolation, and cellular phosphatase activity assays

For the re-expression of RNA interference-resistant PGP in stably PGP-depleted murine spermatogonial GC1-spg cells, here referred to as GC1 cells [9], human Pgp^{WT} or Pgp^{D34N} in pcDNA3 were transfected using Lipofectamine 2000 (Invitrogen); tdTomato (Clontech) was expressed in control shRNA cells to correct for potential effects of exogenous protein expression. Cells were trypsinised, resuspended in fractionation buffer [30 mM triethanolamine, 1 mM $MgCl_2$, 150 mM NaCl, 10 µg/mL aprotinin, 10 µg/mL leupeptin, 1 mM pepstatin, 1 mM 4-(2-aminoethyl)benzenesulfonyl fluoride; pH 7.5], and lysed on ice by repeatedly drawing into a syringe with a 25G × 5/8 inch needle. Insoluble material was removed by centrifugation at 10,000 × *g* for 12 min at 4°C. For the isolation of red blood cells, ~600 µL blood from $Pgp^{flx/flx}$ or $Pgp^{flx/flx}$*; Tie2-Cre*$^{+/-}$ mice was collected into 250 µL acid citrate dextrose buffer. Blood was centrifuged at 410 × *g* for 5 min at room temperature, and the pellet was washed three times in 500 µL 0.9% NaCl. Red blood cells were lysed by freezing and thawing in liquid nitrogen. After the addition of 1 mL fractionation buffer, insoluble material was removed by centrifugation at 10,000 × *g* for 12 min at 4°C. To remove haemoglobin (which interferes with malachite green-based free phosphate detection), cell lysates were fractionated at 4°C by size exclusion chromatography on a Superdex 200 10/300 GL column operated on an ÄKTA liquid chromatography system (GE Healthcare). Proteins were eluted in fractionation buffer with a flow rate of 0.75 mL/min and collected in 24 fractions of 1 mL each. Fractions were analysed for the presence of PGP by Western blot [9]. PGP activity was assayed in 10 µL of each fraction after 60 min preincubation at 22°C in fractionation buffer containing 5 mM DTT (total assay volume, 100 µL). The reaction was started by the addition of 0.5 mM PG and stopped after 5.5 min by adding 100 µL malachite green solution (Biomol Green; Enzo Life Sciences). Released phosphate was determined by measuring A_{620} and extrapolating the values

to a phosphate standard curve. To measure cellular phosphatase activity against *p*NPP, 100 μL of each fraction were preincubated for 30 min at 22°C in the presence of 1 mM DTT. The reaction was started by the addition of 3.5 mM *p*NPP, and *p*NP generation was determined after 1 h by measuring A_{405}.

Histology and immunohistochemistry

For thin sections (1 µm), yolk sacs were fixed for 1 h in 4% (w/v) *para*-formaldehyde (PFA), dehydrated in acetone, embedded in Epon (Epoxy Embedding Medium Kit) and stained with 0.1% methylene blue/1% borax. For immunohistochemistry, uteri, embryos or yolk sacs were dissected, fixed in 4% PFA, cryoprotected by incubating in 20% (w/v) sucrose/PBS, and frozen in Tissue-Tek O.C.T. compound (Fisher Scientific). Endogenous peroxidase activity in the cryosections (5 µm) was quenched for 10 min with 0.3% (v/v) H_2O_2, sections were permeabilised and blocked in blocking buffer [10% (v/v) normal goat serum in PBS, supplemented with 1% (v/v) BSA and 0.1% (v/v) Triton X-100], and incubated with rabbit polyclonal α-PGP antibodies or rat monoclonal α-CD31 antibodies (BD Biosciences) and biotinylated goat α-rat/α-rabbit secondary antibodies (BD Biosciences). Bound antibodies were detected using the Vectastain Elite ABC kit, and specimens were developed with the DAB Peroxidase Substrate Kit (both from Vector Laboratories). Whole-mount immunohistochemistry of E11.5 embryos was performed accordingly, except that endogenous peroxidase activity was quenched for 1 h with 0.3% H_2O_2. Allantois vascularisation was analysed by explanting freshly isolated allantoides in 12-well chambers (ibidi) precoated with 10 μg/mL α4β1 integrin (R&D Systems). After overnight spreading, allantoides were fixed with 4% PFA and stained overnight at 4°C with rat monoclonal α-CD31 antibodies. Bound antibodies were detected with alexa 488-labeled goat α-rat secondary antibodies (Invitrogen).

SUPPLEMENTARY FIGURE LEGENDS

Supplementary Figure S1. Loss of PGP phosphatase activity does not primarily affect (extra)embryonic vascularisation. (**A**) RNA *in situ* hybridisation analysis of embryos. Markers: *Flk1*, *Flt1*: endothelia; *Sm22*: vascular smooth muscle cell layers. (**B**) Whole-mount CD31 immunohistochemical analysis of embryonic vasculature. The boxed areas magnified below show intersomitic vasculature and sprouting in the neural tube. (**C**) Analysis of yolk sac vascularisation by whole-mount immunohistochemistry (*upper panels*) and by histochemistry of transverse sections (*lower panels*). (**D**) Whole-mount immunohistochemistry of vascularisation in allantois explants. (**E**) Steady-state protein tyrosine phosphorylation levels in PGP-deficient embryos. Whole embryo lysates (*n*=2 embryos per genotype) were immunoblotted with 4G10 α-phosphotyrosine antibodies.

Supplementary Figure S2. Establishment of a fractionation-based cellular PGP phosphatase assay. (**A**), Western blot showing the expression levels of endogenous PGP in murine GC1 cells stably expressing control (ctrl) shRNA or murine (m)PGP-directed shRNA. For add-back experiments, PGP-depleted cells were transfected with RNAi-resistant human (h)PGP^{WT} or $hPGP^{D34N}$. Control cells were expressing tdTomato. (**B**) Protein concentration profiles of GC1 cell lysates fractioned by size-exclusion chromatography. (**C**) Identification of PGP-containing fractions by immunoblotting. The arrowhead indicates a nonspecific (nonspec.) band. (**D**) *p*NPP phosphatase activity assays in cell lysates fractionated by size-exclusion chromatography. *p*NPP dephosphorylation was detected photometrically by generation of *p*NP. Results are mean values ± S.E.M.; *n*=4.

Supplementary Figure S3. PGP phosphatase activity in red blood cells. (**A**) Western blot analysis of endogenous PGP expression levels in whole cell lysates of erythrocytes isolated from *Pgp*$^{flx/flx}$ and *Pgp*$^{flx/flx}$; *Tie2-Cre*$^{+/-}$ mice. (**B**) Protein concentration profiles of red blood cell lysates fractioned by size-exclusion chromatography. (**C**) Identification of PGP-containing fractions by immunoblotting. PGP was undetectable in fractions #1-8 and #18-24. (**D**) PGP phosphatase activity in fractionated erythrocyte lysates was measured with malachite green. Mean absorbance values ± S.E.M. in the individual fractions are shown; n=5.

Supplementary Figure S4. Analysis of apoptosis in E8.5 embryos. DNA fragmentation in *Pgp*$^{WT/WT}$ (left panel) and *Pgp*$^{D34N/D34N}$ (right panel) embryos was visualized by whole-mount TUNEL staining. Confocal microscopic images are shown. The upper panels show the respective embryo, the lower panels show the allantois of the same specimen.

Supplementary Figure S5. Glycerolipid profiling of E8.5 embryos by LC/MS. After data preprocessing, 494 of the 3780 aligned compounds were significantly different in total lipid extracts of pooled *Pgp*$^{WT/WT}$ and *Pgp*$^{D34N/D34N}$ embryos ($p \leq 0.02$, fold change ≥ 3). Orthogonal partial least square discriminant analysis of all aligned compounds was performed to filter out statistically relevant lipid compounds that discriminate between *Pgp*$^{WT/WT}$ and *Pgp*$^{D34N/D34N}$ embryos. Lipid features that were significantly down- or upregulated in the mutant embryos are marked with a dotted or dashed line box, respectively. By comparing accurate masses of the molecular ions with the Metabolite and Tandem MS Database Metlin, the two up-regulated PGP lipid markers were identified as DG-16:0-18:0 and DG-18:0-18:0.

Supplementary Figure S6. TG and PtdCho profiling of E8.5 embryos by LC/MS. Glycerolipids were analysed in total lipid extracts of 10 pooled E8.5 embryos. Data shown are mean values of triplicate determinations ± S.D.

SUPPLEMENTARY TEXT

Normal vascular development in PGP-deficient embryos and -extraembryonic tissues

Comparison of the vasculature of E10.5 *Pgp*$^{WT/WT}$ and *Pgp*$^{DN/DN}$ embryos showed that vessels were formed, large vessels had muscular walls, all endothelia were *Flk1*-positive, efficient sprouting into the neural tube occurred, and arteriovenous vessel specification appeared normal (**Supplementary Fig. S1A**). Despite the developmental delay of E11.5 *Pgp*$^{D34N/D34N}$ embryos, intersomitic vasculature was present and sprouting into the neural tube was seen (**Supplementary Fig. S1B**). We did not observe PGP-dependent differences in yolk sac vascular plexus formation at E8.5, nor in vascular density, branching, or organisation into vessels of different calibres in E10.5 yolk sacs (**Supplementary Fig. S1C**). Furthermore, transverse sections showed that blood islands were properly formed and filled with blood cells at E10.5. We sometimes noted endodermal hyperproliferation around the blood islands of E10.5 *Pgp*$^{D34N/D34N}$ yolk sacs (**Supplementary Fig. S1C**). This can be a compensation response to embryo starvation due to insufficient transplacentar supply [55], and may therefore represent a secondary effect caused by embryonic growth impairment. Allantois explants from E8.5 *Pgp*$^{WT/WT}$ and *Pgp*$^{D34N/D34N}$ embryos did not show obvious differences in CD31-positive structures (**Supplementary Fig. S1D**). Given the important role of protein tyrosine phosphorylation events for vascular development and the previously observed involvement of PGP (AUM) in EGF-induced signal transduction [9, 11], we also examined protein tyrosine phosphorylation levels. However, we did not detect obvious alterations in E10.5 *Pgp*$^{D34N/D34N}$ embryos compared to their wildtype counterparts under these steady-state conditions (**Supplementary Fig. S1E**).

SUPPLEMENTARY REFERENCE

55 Tasaki, T. *et al.* UBR box N-recognin-4 (UBR4), an N-recognin of the N-end rule pathway, and its role in yolk sac vascular development and autophagy. *Proceedings of the National Academy of Sciences of the United States of America* **110**, 3800-3805, doi:10.1073/pnas.1217358110 (2013).
